# The truth is hidden in the details

**DOI:** 10.1007/s00709-024-01963-w

**Published:** 2024-06-18

**Authors:** Peter Nick

**Affiliations:** Joseph Gottlieb Kölreuter Institute for Plant Sciences, Karlsruhe Institute of Technology, Karlsruhe, Germany

Science is a collective method to search for truth. If truth were obvious, we would not need science. As Xenophanes put it, more than 2500 years ago: “The gods did not reveal, from the beginning, all things to us; but in the course of time, through seeking, we may learn, and know things better” (in the translation by Popper et al. 1998). Our search is guided by guesses, called explanations. To what extent an explanation approximates truth is often judged by its predictive power. When an explanation allows us to derive implications and when we later find these implications confirmed by experiments, we tend to believe that we steer into the right direction. However, empiry is not the only criterion for the quality of an explanation—we also apply esthetical criteria. A simple, elegant explanation is usually preferred over a version that is complex and requires additional assumptions. This principle, attributed to William of Ockham (according to Poncius 1639): *pluralitas non est ponenda* *sine necessitate* “do not use complexity without necessity,” later known as Ockham’s Razor, is intuitive, but it is uncoupled from empiry. In biology as an empirical science, an explanation, as elegant and parsimonious it may be, will need to go, if experimental evidence speaks against it. Two contributions to the current issue show impressively that “obvious” is not a synonym for “true.”

Soil salinity is an emerging issue in agriculture, due to climate change. Rising sea levels spoil fertile land along the coastlines, and artificial irrigation leaves behind a trace of salt once the water has evaporated. Crop plants able to cope with salinity have acquired, therefore, increasing attention. Quinoa, originating from the Andean salt deserts, is one of these crops with superior salt resistance. It can accumulate sodium in the older leaves and shed those later, thus protecting the younger leaves. The leaf epidermis of this plant is covered with bladder-like cells, and the morphology of these cells along with the salt tolerance of quinoa has led to the idea that these cells are involved in the secretion of sodium. This idea is obvious, and it clearly meets the criteria of Ockham’s Razor. However, it is wrong, as shown in the work by Palacios et al. (2024) in the current issue. They use a combination of microscopical anatomy with advanced imaging including confocal RAMAN microscopy, environmental scanning spectroscopy, and energy-dispersive X-ray analysis to actually follow ionic composition and content. They can demonstrate that it is not sodium but potassium that is accumulating in the bladder cell with a clear gradient increasing on the path through the stalk to the bladder cell. They show further that the main sink for sodium is the stem parenchyma and EDX analysis reveals that the salt in the stem is forming crystals covered by an insoluble calcium oxalate layer, an efficient mechanism to sequester sodium also known from other species (Brizuela et al. 2007). This re-opens the question, what the function of the bladder cells might be. Recent evidence suggests that they are part of herbivore defense (Moog et al. 2023), which is quite far from the obvious, but misleading, correlation between salinity and bladder cells.

A similar pattern of hidden complexity emerges from a comparative study on the sensory structures of mosquito antenna in the contribution by Albergaria et al. (2024) to the current issue. Also here, the background for this study is of considerable societal impact. Hemophagous mosquitoes are important vectors for pathogens. Sensing and locating the host are a crucial element of their lifestyle. Hemophagy has evolved several times independently, which means that there are usually phytophagous relatives. This coexistence sets the stage for comparisons in order to understand what anatomic changes might have enabled the transition to hemophagy. The authors address this by comparing two exclusively phytophagous species of the genus *Toxorhynchites* with the species *Lutzia bigoti*, where the females are hemophagous. Using scanning electron microscopy, they can locate five types of sensilla that appear to be similar over the three species. Sexual dimorphism is also reflected in the abundance and size of some sensilla, probably linked with the necessity of males to track the females by their pheromones. However, as tentative it may seem, authors could not detect a link between sensilla structure and feeding habit. For instance, type-II trichoids allowing females of *Aedes aegypti*, the vector for Dengue Fever, to find oviposition sites, after having sucked blood, are also present in the two phytophagous *Toxorhynchites* species. On the other hand, the hemophagous *L. bigoti* lacks them. Authors conclude that it was not structural changes in the sensilla that enabled the transition to hemophagy. Instead, sensilla apparently respond to olfactory cues that are shared between phyto- and hemophagous mosquitoes. These might be very basic compounds, such as carbon dioxide, that would be valid cues for both plant and vertebrate hosts. Again, a seemingly straightforward hypothesis on the structural base for a biological function turns out invalid.

Both cases are paradigmatic beyond the actual structure–function relationship reported. In both studies, Occam’s Razor gets notched if challenged by scrutiny. On the other hand, the far more complex reality emerging from this scrutiny remains consistent, although new details become visible. In fact, it is the matching details that lend credibility and support to the conclusions drawn from such studies. While Occam’s Razor may be a good start to get into a phenomenon, elegance is rarely in nature, but in the way how we want to see nature. Thus, elegance is not a serious criterion for truth—instead, truth is hidden in the details.
